# Development and Characterization of Hemicellulose-Based Films for Antibacterial Wound-Dressing Application

**DOI:** 10.3390/polym12030548

**Published:** 2020-03-03

**Authors:** Naveed Ahmad, Danial Tayyeb, Imran Ali, Nabil K. Alruwaili, Waqas Ahmad, Atta ur Rehman, Abdul Haleem Khan, Mohammad Saeed Iqbal

**Affiliations:** 1 Department of Pharmaceutics, College of Pharmacy, Jouf University, Sakaka, Aljouf 72388, Saudi Arabia; 2 Department of Pharmacy, Forman Christian College, Ferozepur Road, Lahore 54600, Pakistan; 3 Department of Chemistry, Forman Christian College, Ferozepur Road, Lahore 54600, Pakista; 4 School of Pharmaceutical Sciences, University of Science Malaysia, Pulau Pinang 11800, Malaysia

**Keywords:** antibacterial dressing, hemicelluloses, wound infection, drug delivery

## Abstract

Hemicelluloses are biopolymers with versatile properties for biomedical applications. Herein, hemicellulose (arabinoxylan)-based antibacterial film dressings were prepared and characterized. Arabinoxylan was isolated from psyllium husk. Blank and gentamicin-loaded films were prepared by the solvent cast method using glycerol as the plasticizer. The appropriate composition of the films was obtained by varying the amounts of arabinoxylan, glycerol, and gentamicin. The films were found to be transparent, smooth, bubble-free, flexible, and easily peelable with 2% to 3% arabinoxylan. They had uniform thickness and swelled up to 60% of their initial size. The mechanical properties and water vapor transmission rate through the films were found to be suitable for wound-dressing application. Fourier transform infrared spectroscopy (FTIR) analysis confirmed drug–film compatibility. In an in vitro release study, more than 85% of the gentamicin was released from the films in 12 h. The antibacterial activities of the gentamicin-loaded films were found to be close to the standard gentamicin solution. The films were found to be cytocompatible in cell viability assay. These results suggested that hemicellulose-based films are promising materials for the dressing of infected wounds.

## 1. Introduction

A wound is a disruption in the anatomy and physiology of skin that may be caused by physical, chemical, or thermal injuries, or by pathological conditions [1,2]. In order to repair the disrupted skin function, the wound-healing process starts immediately after development of the wound. This is a complex process that involves a series of coordinated events including homeostasis, inflammation, proliferation, remodeling, and scar maturation [3,4,5,6]. The wound-healing process may be further complicated by various factors such as diabetes mellitus, bacterial contamination, poor wound care, and patient lifestyle [7,8]. The immune response of the host and the wound-healing process are compromised in an infected wound containing 10^6^ CFU/mL of bacteria, which not only delays the wound-healing process but can also lead to life-threating consequences [9,10,11,12]. Therefore, wound dressings are applied on the wound area to protect the wound from bacterial contamination, deliver antibiotics, absorb wound exudate, and provide a moist environment (required for ideal healing) [2,13,14]. However, the application of conventional wound dressings (gauze, pads, and cotton) in combination with topical antibiotic formulations result in leakage of the drug, messiness, and dryness of the wound area, which lead to poor wound healing and patient compliance. Therefore, there is a need to develop a wound dressing that can prolong the residence time of the antibiotics in the infected wound, absorb exudate, and maintain a moist environment around the wound area [15]. 

Modern wound dressings (film wafers, hydrogels, and sponges) are designed to overcome the limitations of conventional wound dressings. The design and application of these dressings has been comprehensively reviewed [2,16], where it has been emphasized that the dressing materials must be biocompatible, exudate absorbing, allow water vapor transmission (WVTR), and possess antibacterial properties [16,17]. A simple method of preparation, excellent WVTR, transparency, and cost effectiveness are the attributes of good film dressings [18,19]. 

Numerous synthetic and natural polymers and combinations thereof have been explored for making film dressings. Among the natural polymers chitosan, gelatin, alginate, pectin, and cellulose have shown promise for this purpose [12,13,15,20,21,22]. However, insufficient mechanical strength, stability, poor exudate-absorbing capacity, and adherence with wound site are the major limitations associated with natural polymer-based films [16]. In order to overcome these limitations, there is a need to explore more biopolymers for such applications. Hemicelluloses isolated from plant materials, being highly biocompatible, biodegradable, and cost effective, are gaining popularity for use in drug delivery and tissue engineering [23,24]. They are the second most abundant biomass on earth. Arabinoxylan (AX) from psyllium (*Plantago ovata*) seed husk, one of the most studied hemicelluloses, presents a great promise for drug delivery formulations [25,26,27,28]. Over past two decades, this polymer has been extensively characterized with regard to its structure and composition [26,29,30,31,32]. It possesses excellent stability, water-absorption capacity, and excellent film-forming properties, which make it a promising candidate for antibiotic delivering dressings.

In the present work, AX-based film dressings have been prepared for the delivery of antibiotics, incorporating gentamicin (GM) as a model drug, to the infected wound. In a pre-formulation study, optimal amounts of AX and glycerol required to prepare films have been determined. The prepared films were characterized by various analytical techniques and tested, in vitro, for their effectiveness as wound dressing. 

## 2. Materials and Methods

### 2.1. Materials

*Plantago ovata* (PO) seed husk as source of hemicellulose (Arabinoxylan) was purchased from a local market. Glycerol, disodium hydrogen phosphate, dipotassium hydrogen phosphate, dihydrogen potassium phosphate, sodium chloride, potassium chloride, Mueller–Hinton agar, and sodium hydroxide were from Sigma-Aldrich (St. Louis, MO, USA). *Pseudomonas aeruginosa* (ATCC 9027), *Escherichia coli* (ATCC 8739)*,* and *Staphylococcus aureus* (ATCC 25923) were obtained from the American Type Culture Collection (ATCC, Manassas, WV, USA). Gentamicin (antibiotic) was a gift from Venus Pharma, Lahore, Pakistan. Distilled water was used throughout this work.

### 2.2. Isolation of Arabinoxylan

Hemicellulose (Arabinoxylan (AX)) was extracted from PO seed husk by slight modification of a previously reported method [32]. In brief, 10 g of husk was soaked in 1000 mL of distilled water for 24 h; then, the upper insoluble layer was discarded. Then, the thick gel obtained was vacuum filtered using muslin cloth and the filtrate was then concentrated by using rotary evaporator (R210, BÜCHI AG, Flawil, Switzerland) at 35–40 °C. Then, the concentrated gel obtained after evaporation was dried on a polythene sheet in air until a constant weight was achieved. 

### 2.3. Preparation of AX Films

Blank and GM-loaded AX films were prepared by the solvent-casting method [33]. The blank AX films were prepared by using different amounts of AX and glycerol (Table 1). Briefly, AX powder was added in water (90 mL) and stirred (500 rpm) at room temperature until a homogeneous gel formed. To this, glycerol was added, the volume was made to 100 mL with water, and the mixture was sonicated to remove bubbles. The gel (~33 g) thus obtained was poured into plastic 9.5-cm petri dishes to cast the blank films, which were subsequently dried at 40 °C to a constant weight. For the preparation of GM-loaded films, 1 mL of GM solution (100 mg·mL^−1^) was added to casting gel obtained above after the addition of glycerol and stirred (500 rpm) at room temperature to obtain a homogeneous gel. The films were cast and dried as above. The composition of blank and GM-loaded films along with their codes is given in Table 1. The prepared films were tested for their peelability, bubbles, wrinkles, foldability, and transparency.

### 2.4. Physical Assessment of Films

The blank and GM-loaded films were physical examined for their peelability, the presence of bubbles, transparency, odor, and flexibility. 

### 2.5. Characterization of Films

#### 2.5.1. Measurement of Thickness 

The uniformity of the films was determined by measuring the thickness of the films at five different points by using a manual screw gauge. 

#### 2.5.2. Solvent Loss 

The water lost during the film-casting (drying) process was determined by the formula:

$$Solvent\;loss\;(\%)\;=\frac{M_{i\;}{\;–\;M}_{d}}{Mi}\;\times \;100,$$

where *M_d_* is the mass of the dried films and *M_i_* is the mass of the casting gel.

#### 2.5.3. Mechanical Characterization of Films

A Universal Testing Machine (UTM) (LS5, Lloyd Instruments Limited, West Sussex, UK) was used to measure the tensile strength and percent elongation at break. For this, the films were cut into dumbbell-shaped (3 cm × 0.5 cm) strips. The strips were subjected to extension by UTM at a speed of 0.5 cm·min^−1^ until breaking the films. The following equations were used to calculate the tensile strength and percent elongation at break.

$$Tensile\;Strength\;=\;\frac{\;Max.\;load\;at\;break\;}{Transverse\;section\;area}$$

$$Elongation\;at\;break\;\left(\%\right)\;=\;\frac{\;Extension\;of\;length\;at\;rupture\;}{Initial\;length}\;\times 100$$

#### 2.5.4. Determination of Extent of Swelling

The swelling index of the films was determined in a simulated wound exudate environment according to a reported method [34]. Gelatine solution (4% w/v) was used as the swelling medium to simulate the wound exudate environment. Briefly, gelatine solution (20 mL) was poured into petri dishes and left overnight. Discs (~2 cm diameter) cut from the films were placed over the gelatine medium, and an increase in the diameter (as expansion diameter) of the films was recorded at predefined time intervals. The percent swelling of the films was calculated by the formula:

$$Swelling\;(\%)\;=\;\frac{Diameter\;of\;swelled\;film\;-\;Initial\;diameter\;of\;film\;}{Initial\;diameter\;of\;film\;}\;\times \;100$$

#### 2.5.5. Water Vapor Transmission Rate (WVTR)

The transport of water vapors across the films was investigated by a slightly modified reported method [13]. Glass vials with caps having holes (1 cm diameter) were filled with silica gel. The films were mounted on the vial opening, and caps were fitted. The vials were placed a desiccator maintained at 85% and 25 ± 2 °C by using aqueous potassium chloride solution (27%). After predefined time periods, the vials were removed and weighed to determine the gain in weight. A glass vial with an aluminum foil without the film in it was used as control. The following equation was used to calculate the WVTR.

$$WVTR\;=\;\frac{\left(Wt-Wi\right)\;\times \;t}{A}$$

where *W_t_* is the weight of the vial at time *t*, *Wi* is the initial weight of the vial, *t* is the time (h), and *A* is the exposed area (m^2^) of the film. 

#### 2.5.6. Fourier Transform Infrared Analysis

Fourier transform infrared (FTIR) analyses of GM, AX powder, glycerol, AXF2.5, and AXFD2.5 were performed using an FTIR spectrometer with attenuated total reflectance (Cary 630, Agilent, Santa Clara, CA, USA). The FTIR spectra of the samples were recorded over the wavenumber range of 4000 to 650 cm^−1^.

#### 2.5.7. Thermal Analysis 

Thermogravimetric analyses (TGA) and differential scanning calorimetry (DSC) of the AX powder, blank, and GM-loaded films were performed using a thermal analyzer SDT, Q-600 (T.A. Instruments, New Castle, DE, USA). The samples (2–3 mg) were heated from ambient to 500 °C at a heating rate of 10 °C·min^−1^ under nitrogen flow (100 cm^3^·min^−1^). 

#### 2.5.8. Scanning Electron Microscopy

Scanning electron microscope (SEM) was used to observe the distribution of the GM particles in the films. For this purpose, small pieces of AXF2, AXF3, AXFD2.5, and AXFD3 films were gold coated, and micrographs were captured at 500× magnification using SEM (FEI, Inspect S50, Brno, Czech Republic). 

### 2.6. In Vitro Drug Release Study

The release of GM from the loaded AX films was studied using a Franz diffusion cell (DHC-6T Transdermal System with a DHC-800 system controller from Logan Instruments Corp, Somerset, NJ, USA). The donor and receptor compartments were separated by a cellulose acetate membrane (0.45 µm). Phosphate-buffered saline (PBS) at pH = 7.4 was used as the release medium (10 mL) in the receptor compartment, and the temperature was adjusted to 37 °C. The loaded films were cut into small discs weighing approximately 100 mg and carefully placed on the membrane in the donor compartment. Then, the donor and receptor compartments were clamped and covered to prevent the films from drying. The amount of the GM released was determined by withdrawing an aliquot (1 mL) of the release medium from the receptor compartment at predefined intervals of time (using auto-sampler) and measuring the absorbance at 235 nm by a UV–Vis spectrophotometer (Jenway, Staffordshire, UK). The standard calibration curve of standard GM solutions (0.062, 0.125, 0.25, 0.5, and 1 mg/mL) was used to calculate the GM concentration in the release medium. The drug-release profile was generated by plotting cumulative release as a function of time. To explain the kinetics and mechanism of drug release, zero order, first order, Higuchi, Korsmeyer–Peppas, and Hixon Crowell models were employed on drug release data.

### 2.7. Antibacterial Activity

The antibacterial activity of the loaded films was determined against Gram-positive (*S. aureus*) and Gram-negative (*E. coli and P. aeruginosa*) bacteria using by Kirby–Bauer disk diffusion test [35]. The inocula (1 × 10^6^ CFU·mL^−1^) were prepared by culturing bacterial strains in separate flasks containing 50 mL of sterilized Mueller–Hinton broth (MHB) at 37 °C for 24 h. The sterilized Mueller–Hinton Agar (MHA) was poured into three petri plates and allowed to solidify. The 100 µL inocula were seeded into the respective petri plate. The films were cut into small discs (0.5 cm diameter) containing GM (0.16 µg) and placed on a filter-paper sport. A filter paper disc (without the film on it) was spotted with 10 µL of standard GM solution (containing 0.16 µg GM) to prepare a positive control. The GM-loaded film, the blank film (AXF2.5), control positive, and control negative (blank filter paper) were placed on the petri pates inoculated with the organism. These plates were incubated at 37 °C for 24 h. The antimicrobial activity was determined by measuring the zone of inhibition (ZOI) in mm on the plates. All these experiments were carried out in triplicate, and the mean ± SD was reported.

### 2.8. Cell Viability Assay

The cytotoxicity of AX films was investigated by performing alamarBlue® assay (Invitrogen, Carlsbad, CA, USA) on baby hamster kidney cells (BHK-21, ATCC^®^, Manassas, VA, USA) using an indirect contact method (according to ISO-10993 standard) [13]. The culture medium was prepared by adding 10% fetal bovine serum (FBS) and 1% antibiotic (penicillin-streptomycin) in Dulbecco’s modifed Eagle’s medium (DMEM). The cells were incubated in CO_2_ atmosphere at 5% RH and 37 °C to achieve 80% confluence. The AX films (50 mg pieces) were sterilized by UV light (45 mins for each side) and then incubated with culture medium for 24 h to obtain extracts of films. The cells were suspended in culture media, and approximately 1 × 104 cells were seeded in each well of 96-well cell culture plates. The plates were incubated for 24 h, and then medium was replaced with 100 µL AX films extracts in sample wells and fresh medium in control wells. Then plates were again incubated for 24 h, and alamarBlue® assay (Invitrogen, Carlsbad, CA, USA) was performed to estimate the percentage of viable cells by measuring the optical density of the control and sample wells using microplate reader at 570 nm [33]. 

### 2.9. Statistical Analysis

The experimental data were statistically analyzed by performing one-way ANOVA followed by Tukey’s test and nonparametric *t*-test using GraphPad Prism (V 5.02, GraphPad Software, Inc., San Diego, CA, USA). The *p* values less than 0.05 were considered as the significant difference.

## 3. Results and Discussion 

### 3.1. Isolation of Arabinoxylan

The gel obtained from psyllium seed husk is mainly composed of AX (M_W_ of 364,470 g/mol, arabinose 74.8% and xylose 23.2%) [32]. The air-dried gel of husk formed highly transparent thin films. The yield value of AX from psyllium seed husk was calculated to be 20.3%. Then, the transparent AX sheets were ground to obtain whitish AX powder that was used in further experiments. 

### 3.2. Preparation and Physical Assessment of AX Films

The pure AX formed transparent thin films (Section 3.1), which often ruptured on peeling off the mold (petri dish). They also did not absorb a sufficient volume of exudate from the wound area. In order to have thicker films, the weight of the AX gel used to cast the films was increased appropriately through hit and trial experimentation. The thickness of the AXF films thus obtained (Table 1 and Table 2) was suitable, but they became more brittle. In order to overcome this problem, the use of glycerol as a plasticizer was considered. Therefore, experiments were performed to find a suitable composition with respect to AX and glycerol (Table 1). These films were assessed for transparency, the presence of bubbles, ease of peelablity from petri, and flexibility. On the basis of the results of this assessment (Table 2), AXF2, AXF2.5, and AXF3 films were selected for the preparation of drug-loaded films. The drug-loaded films (AXDF2, AXDF2.5, and AXDF3) containing 0.1% GM were prepared. The resulting drug-loaded films were found to be transparent and could easily be peeled off the perti dish. The transparency of the films is important, as it allows the physician to observe wound-healing progress.

### 3.3. Characterization of AX Films

#### 3.3.1. Film Thickness

Film designed for wound healing and drug delivery application should be of uniform thickness to ensure the homogeneous distribution of the drug and uniform mechanical properties. The films’ thickness is mainly governed by the distribution of the solid (polymers, drug) in the film, the amount of gel used for casting films, and the flatness of the mold. In order to ensure a uniform distribution of solids, the casting gel was homogenized before pouring it into the petri dish. For this, equal amount of gels was used to cast the films, and the gels were dried on a flat surface. The results are given in Table 3 and depicted in Figure S1. These results indicate that the thickness of the films increased with the increasing amount of AX; the thickness of the GM-loaded films was slightly greater than that of the corresponding blanks, as expected.

#### 3.3.2. Solvent Loss

It is important to estimate the solvent (water) loss during the film-drying process, as it provides information on the solvent required to hydrate films to their original shape (casting gel) and the amount of exudate that can be absorbed by the film before it completely transforms into free-flowing gel that can be washed from the wound site. As the amount of gel used for casting films was constant for all the formulations, the solvent will mainly depend upon the amount of solid in film formulation. The results of the solvent loss study are given in Table 3 and depicted in Figure S2. These results indicated that solvent loss during the drying process decreased with the increasing amount of AX with a fixed amount of GM in film formulations. This suggests that approximately 30 g of water/exudate will be required to convert the film into a free-flowing gel.

#### 3.3.3. Mechanical Characterization

Tensile strength and percentage elongation at break were measured using UTM. The tensile strength of films for application on skin should be in the range 2.5–16 N·mm^−2^, whereas the elongation at break should be >70% [20]. 

The results of the mechanical testing are provided in Table 3. The blank and GM-loaded films prepared by using 2% AX (AXF2 and AXDF2) were damaged immediately after the start of experiments and their results could not be recorded; whereas the films AXF2.5, AXDF2.5, AXDF3, and AXDF3 exhibited tensile strength and elongation at break in the acceptable range [13,33]. Moreover, it was observed that the tensile strength of the films significantly increased with increasing AX content, indicating that higher AX content results in the formation of stronger films. Similarly, the tensile strength of the films also slightly increased with the incorporation of GM. Several factors can contribute to mechanical strength of the films, the most important being the structure and degree of polymerization of the polymer. The use of plasticizer also enhances the flexibility (elasticity) of the films [9]. Therefore, the good mechanical properties of these films can be attributed to the high degree of polymerization of the AX and the use of glycerol as a plasticizer. Based on these results, it can be concluded that the films with a composition of AXF2.5 and AXF3 were mechanically strong enough for application as wound dressing. 

#### 3.3.4. Swelling Index of the Films

**Figure 1:**
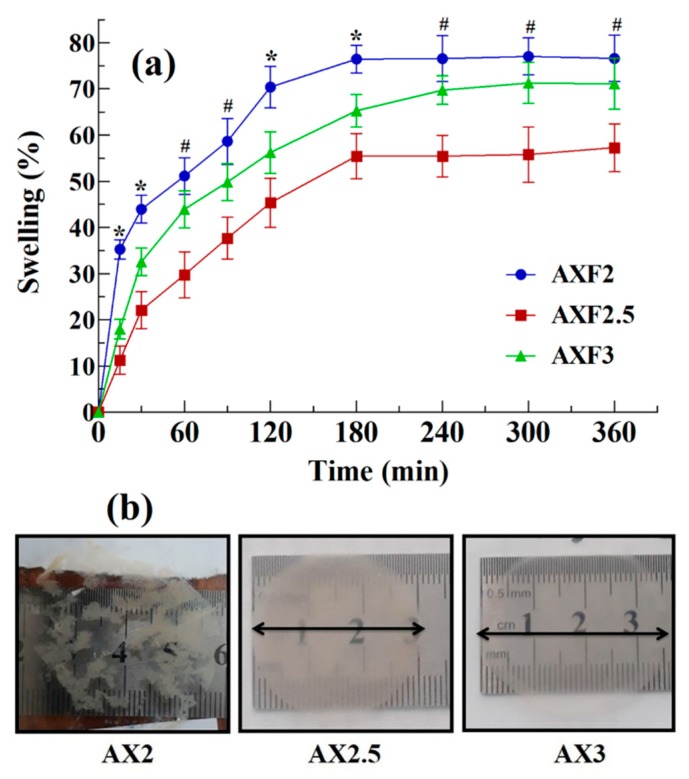
Swelling of the AX films (**a**) swelling index (mean ± SD, n = 3), the asterisks (*) show the significant difference among all three AX films and the hashes (#) show the significant difference of AXF2.5 from AXF3 and AXF2, (**b**) images films after 5 h of swelling.

The results the swelling study of the films in a simulated wound environment (4% gelatin) are depicted in Figure 1a. The AXF2 films exhibited rapid swelling up to 35% in 15 min, but these films also started rupturing immediately. The rupturing of AXF2 films indicated their poor mechanical strength (as discussed Section 3.3.3). An ideal film dressing should remain intact at the wound surface and should be easily removable. Therefore, AXF2 was not considered suitable for wound dressing application. On the other hand, the swelling of films containing higher amounts of AX (AXF2.5 and AXF3) was slower as compared with AXF2. Although these films swelled slowly, the swelling was uniform in all directions and films did not rupture. AXF2.5 and AXF3 reached equilibrium swelling of approximately 55% after 3 h and approximately 72% after 5 h, respectively. The images of the films taken after 5 h of swelling study are shown Figure 1b. It is clear from these images that the AXF2 sample is completely ruptured, whereas the samples of AXF2.5 and AXF3 were intact even after 6 h.

Swelling is an important criterion for the selection of the films for wound dressing and drug delivery applications because when films are applied on a wound, they absorb the wound fluid and expand [36]. This swelling will allow the film to absorb exudate and diffuse (release) incorporated drug. Excessive swelling at the wound surface may result in the conversion of films into free flowing gel that will flow off from the surface of the wound. Therefore, an ideal film should absorb the exudate from the wound surface, provide a moist environment for proper wound healing, and not form free-flowing gel. The results of the swelling study indicated that AXF2.5 and AXF3 were the most suitable compositions for wound-dressing application, as they remained intact in the simulated wound environment. Moreover, these films absorbed 55% and 72%, respectively, which are exudate values that were much less than the solvent required to convert films to free-flowing gel as found in the solvent loss study.

#### 3.3.5. Water Vapor Transmission Rate (WVTR)

An effective wound dressing should not only absorb the exudate from the wound but also transfer moisture to allow the exchanges of gasses. The results of the WVTR test of the films under investigation are given in Table 3. The WVTR values for AXF2, AXDF2, AXF2.5, AXDF2.5, AXF3, and AXDF3 were found to be 1915 ± 135, 1842 ± 170, 1747.4 ± 121, 1631.6 ± 130, 1757.9 ± 130, and 1665.3 ± 155 g/m^2^/24 h, respectively. These results suggest that the WVTR decreased with increasing the AX content and the addition of GM, which can be attributed to the increase in thickness of the film. According to the literature, the WVTR values for normal and injured skin are 204 and 280–5100 g·m^−2^ per 24 h, respectively [33]. The WVTR of commercially available wound dressings usually range from 100 to 3300 g·m^−2^ per 24 h. The WVTR of the films prepared in this study ranged from 1600 to 2000 g·m^−2^ per 24 h, which is near the ideal values (2000 to 2500 g/m^2^/24 h) and thus exhibit the potential of these films for wound-dressing application [33]. 

#### 3.3.6. Fourier Transform Infrared Analysis

**Figure 2:**
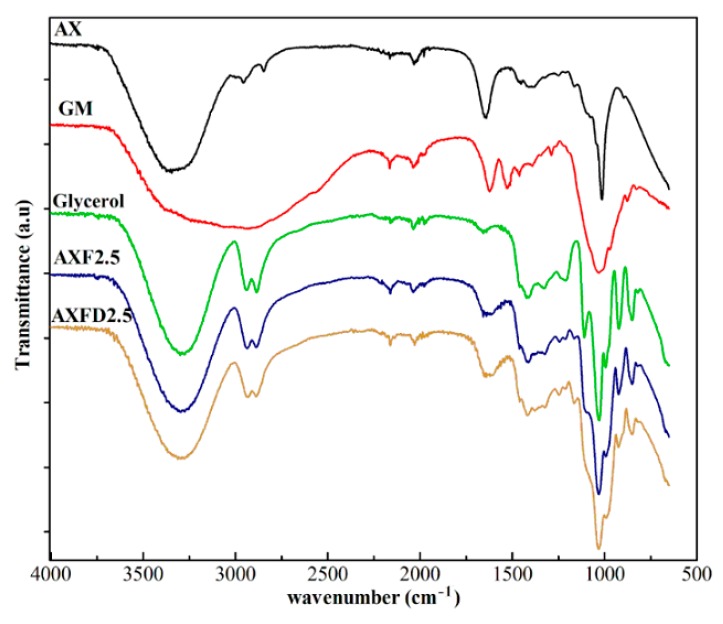
Fourier transform infrared (FTIR) spectra of arabinoxylan (AX), gentamicin (GM), glycerol, blank film (AXF2.5), and GM-loaded film (AXDF2.5).

The FTIR spectra of the GM, AX powder, glycerol, AXF2.5, and AXDF2 are shown in Figure 2. The purpose of FTIR analysis is to confirm the purity of the raw materials and investigate the compatibility of the GM with film components. 

The FTIR spectrum of AX showed υOH at 3343 cm^−1^, υCH at 2955 cm^−1^, absorbed moisture at 1640 cm^−1^, δCH_2_ at1451 cm^−1^, δCH at 1373 cm^−1^, δC–O–C at 1034 cm^−1^, and polymer backbone at 888 cm^−1^, respectively [30,32]. The spectrum of GM showed characteristic peaks of υOH, amide A at 3210 cm^−1^, amide II symmetric stretch at 1621 cm^−1^, amide I stretch at 1526 cm^−1^, δC–O–C at 1030 cm^−1^, respectively [36]. The FTIR spectrum of glycerol exhibited peaks at νOH at 3282 cm^−1^, and νCH_2_ s+as and νCH s+as, at 2940 and 2875 cm^−1,^ νC=C at 1652 cm^−1^, δ–C–H at 1458 cm^−1^, =C–H (cis-) bending at 1416 cm^−1^, δCH at 1321 cm^−1^, C–O stretching at 1236 cm^−1^, C–O stretching or –O–CH_2_–C, ν(C–C) at 1109 cm^−1^, + δCH + νC–C at 1038 cm^−1^, νC–C at 986 cm^−1^, and CH_2_– rocking at 704 cm^−1^, respectively [37].

The FTIR spectrum of the blank (AXF2.5) and the drug-loaded films (AXDF2.5) exhibited all the characteristic peaks of AX and glycerol at unshifted wavenumbers. However, the characteristic peak of the GM was not clearly observed in the spectrum of AXDF2.5 due to the lower GM concentration caused by uniform dispersion into the film matrix and masking the effects of AX and glycerol [20]. Moreover, the absence of any new peak in the spectra of AXF2.5 and AXDF2.5 confirmed the compatibility of the drug–film materials [12]. 

#### 3.3.7. Thermal Analysis 

**Figure 3:**
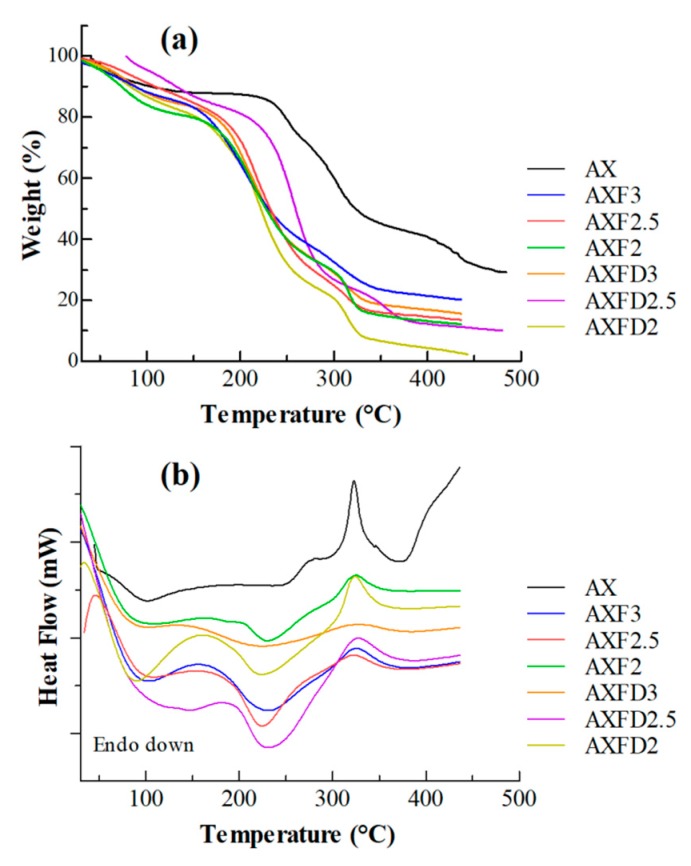
Thermal analysis of AX, blank films, and GM-loaded films, (**a**) thermogravimetric analyses (TGA) and (**b**) differential scanning calorimetry (DSC).

Thermogravimetric analyses of the blank and GM-loaded AX films were performed to investigate the thermal stability of the prepared films. The TG curves of pure AX, blank AX films, and GM-loaded films are shown in Figure 3a. 

The TG curve of the AX shows its three-step degradation. In the first step (ambient to 120 °C), an 11.5% weight loss occurred, which may be attributed to loss of moisture content. In the second step (230 to 335 °C), a 40.5% weight loss occurred that was due to decomposition of AX into smaller fragments. In the third step (above 410 °C), a 12% weight loss was observed that might be due to the complete decomposition of AX into pyrolytic products. The residual weight at 500 °C was 28%, which is most probably the ash content of materials used. These are in close agreement with previous reports [26]. Similarly, the blank and GM-loaded films exhibited three-step thermal degradation, but the onset temperatures of the second and third steps of films were lower as compared to that of AX (Table S1). This change in onset temperature appears to be due the presence of glycerol (decomposes at approximately 160 °C) [38]. The onset of the second and third steps increased with the AX content of the films, which clearly supports the argument that the lowering of the decomposition temperature of the film was due to the presence of glycerol. Moreover, the moisture contents of the films containing glycerol were higher compared to that of AX, and the residue of films at 450 °C was lower than that produced by AX. The thermal degradation behavior of the GM-loaded films was similar to that of the blank films. The residue generated by the GM-loaded films was slightly higher than that produced by the blank, indicating the additional ash content in in the GM used.

DSC curves of pure AX, blank AX films, and GM-loaded films are shown in Figure 3b. The DSC of pure AX exhibited an endotherm (approximately 60–110 °C) due to the release of entrapped water and exotherm (at approximately 325 °C) corresponding with the decomposition of AX [39]. On the other hand, the DSC of blank AX films and GM-loaded films showed two endotherms at approximately 60–130 °C and 190–270 °C, which were attributed to presence of entrapped water and glycerol, respectively [13,40]. Moreover, the exothermic peak of the AX was also observed in DSC of AX films at approximately 325 °C. The DSC curves of the GM-loaded films were similar to those of the blank films with an additional endotherm due to the presence of GM at approximately 250 °C (melting), indicating the compatibility of GM with the components of the films.

#### 3.3.8. Scanning Electron Microscopy

**Figure 4:**
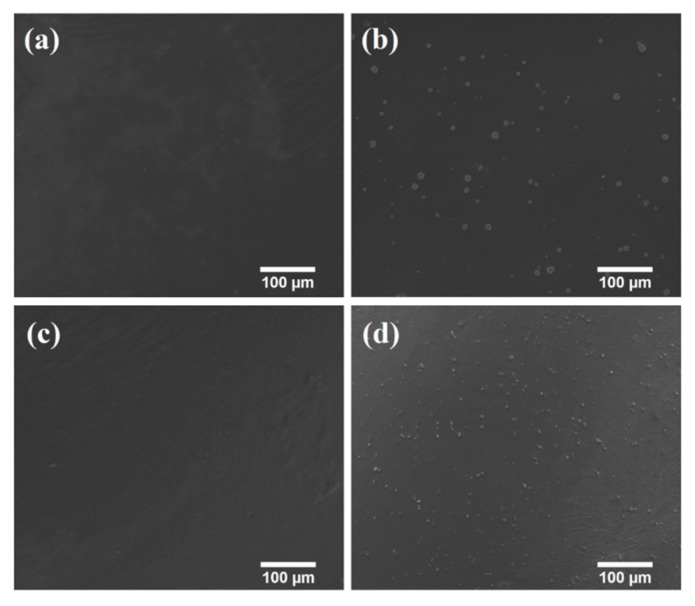
SEM micrographs of (**a**) AXF2.5, (**b**) AXFD2.5, (**c**) AXF3, and (**d**) AXFD3 at 500× magnification.

Scanning electron microscopy (SEM) was used to study the surface morphology and distribution of the drug in the AX films. The micrographs of the AXF2.5, AXFD2.5 AXF3, and AXFD3 are shown in Figure 4. The blank AX films appeared to be smooth, free of voids, and any cracks (Figure 4a,c). On the other hand, drug particles were observed (white spots) in GM-loaded films (Figure 4b,d). Moreover, the size of the drug particles was smaller in AXFD3 as compared to AXFD2.5, which is likely due to the sonication of the AXFD3 casting gel for a longer period of time as compared to AXFD2.5 (required to remove bubble from the film casting gel, as described in Section 2.3). The viscosity of the AXFD3 casting gel was higher compared to AXFD2.5; therefore, a longer sonication time was required to remove the air bubbles from the AXFD3 casting gel. The sonication process disaggregates particle and reduces their size. However, the drug particles were uniformly distributed in GM-loaded films without bubbles and sedimentation and aggregation, which validates the mixing procedure. Thus, the GM-loaded films appear to meet the content uniformity criteria.

### 3.4. Antibiotic Release Study

**Figure 5:**
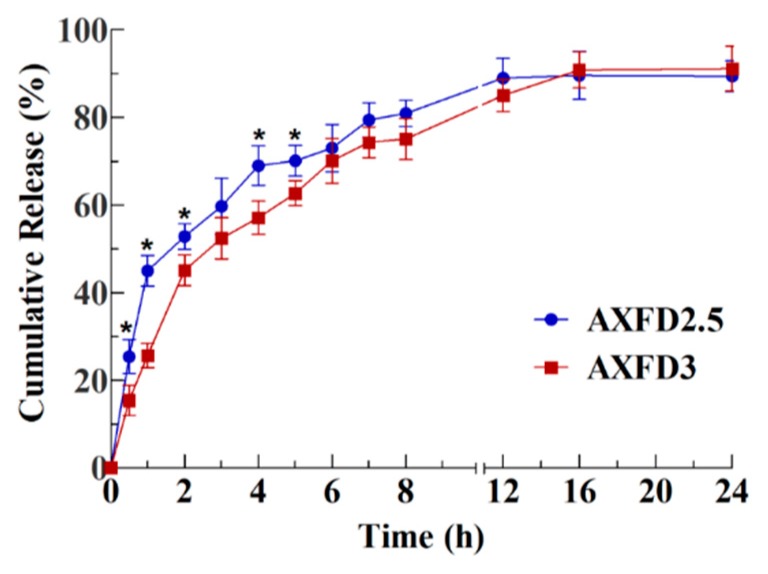
In vitro GM release profile of AX-based films (mean ± SD, n = 3). The asterisks (*) indicate significant differences.

The results of in vitro release study of the selected films (AXFD2.5 and AXFD3) are presented as cumulative release as a function of time in Figure 5. The results of suggest that AXFD2.5 released approximately 45% release in first hour and reached maximum release (approximately 89%) after 12 h, while AXFD3 released approximately 25% of loaded GM in 1 h and exhibited maximum release (approximately 90%) after 16 h. These results suggest that AXFD3 represents a better formulation that is more appropriate for GM delivery at the wound site, as it can maintain the therapeutic GM level for a longer time at the site of the wound. 

The release of drug from a polymeric matrix depends upon the solubility of the drug used, solute diffusion, swelling behavior of the polymer, and degradation of the film matrix [33]. In our study, GM was used as model antibiotic, as it is easily soluble in release medium (PBS). Therefore, a major factor that can affect the release of the drug was the swelling of the film matrix. It was found in swelling experiments that the films with the lowest AX underwent faster swelling (Section 3.3.4). Therefore, AXFD2.5 exhibited faster GM release than AXFD3. The swelling property of the AX films can be considered as the major factor in controlling the release of GM from these films. The optimally prepared films in this study release GM in 12 h, which can further be sustained by cross-linking AX films. 

Different mathematical models were employed on drug release data, and the results are shown in Table 4. These results indicate that the Korsmeyer–Peppas model was the best fitting model because of its highest regression coefficient (R^2^) values (0.911 and 0.979 for AXFD2.5 and AXFD3, respectively). According to this model, the value of diffusion coefficient (*n*) represents the mechanism governing the drug release from the delivery system. For thin films, when the value of ‘*n*’ is between 0.5 and 1.0, drug release is anomalous (non-Fickian) transport [41,42]. The values ‘*n*’ for AXFD2.5 and AXFD3 were 0.511 and 0.651, respectively. These values of diffusion coefficient suggest by non-Fickian transport were the major mechanism governing GM release from AX-based films. These results support the experimental results indicating that drug release from the AX-based films was governed by the swelling of the films.

### 3.5. Antibacterial Activity

**Figure 6:**
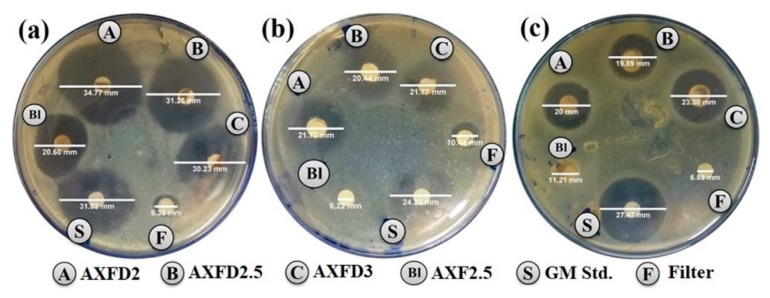
Antibacterial activity of films against (**a**) *S. aureus*, (**b**) *E. coli*, and (**c**) *P. aeruginosa*.

The antibacterial activity of the GM-loaded AX films was tested against *S. aureus*, *E. coli,* and *P. aeruginosa*. These bacterial strains were selected due to their association with wound infection. The zones of inhibition (ZOI) produced by the samples are presented in Table 5 and shown in Figure 6. The result indicates that the control negative (blank filter paper) did not show any inhibitory effect against the tested strains. The blank AX film did not show any significant activity against Gram-negative bacteria (*E. coli strains* and *P. aeruginosa)*, but a significant effect was observed against Gram-positive (*S. aureus*), which can be suggested as being due to the antibacterial activity of pure AX against Gram-positive bacteria, as reported earlier [43]. On the other hand, GM standard solution and GM-loaded films exhibited a significant effect in all the tested strains, as indicated by ZOI values. The values for the drug-loaded films were comparable to those for the GM standard solution in *S. aureus* and *E. coli strains* and lesser than those in *P. aeruginosa*. These results suggest that the antibacterial activity of the GM was retained after incorporation into the films. Therefore, the GM-loaded films show a promise for antibacterial would dressings.

### 3.6. Cell Viability Assay

**Figure 7:**
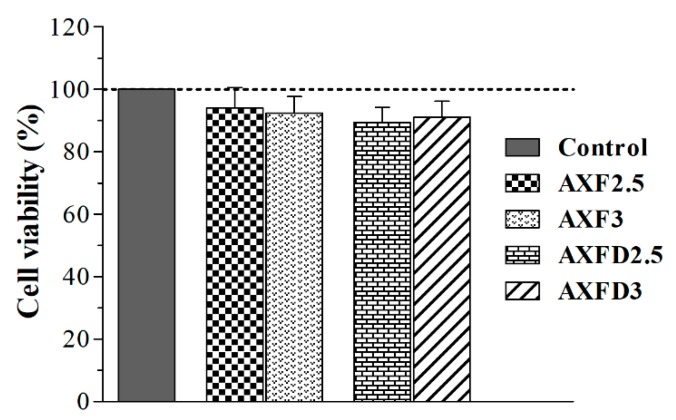
Effect of blank and GM-loaded films on viability BHK-21 cells (mean ± SD, n = 6).

The cytocompatibility of blank and GM-loaded AX films was investigated to assess the safety of films for wound-dressing applications. The indirect contact method was used to investigate cell viability to avoid interference in measuring optical density due to dissolved AX and glycerol [13]. The results of the cell viability assay (Figure 7) revealed that more than 90% cells, treated with extracts of blank and GM-loaded AX films, were viable after 24 h. These results suggest that the tested films are safe for further in vivo investigations as wound dressing application, as a more than 30% decrease in cell viability is considered cytotoxic for dermal applications [44]. 

## 4. Conclusions

Gentamicin-loaded hemicellulose (AX)-based films were successfully developed for the treatment of an infected wound. FTIR and DSC analysis confirmed the drug–film compatibility. The prepared films were transparent, uniform in thickness, and easily peelable. The films allowed an adequate transmission of water vapors. The swelling, mechanical, and release properties of the film can be controlled by the amounts of AX and the plasticizer in films. The release times of gentamicin from the films containing 2.5% and 3% AX were found to be 12 to 16 h; thus, they require a daily change in dressing. The drug-release mechanism appears to be diffusion and swelling controlled. The antibacterial property of gentamicin was not compromised in the formulation of the GM-loaded films. The most prominent feature of the AX-based films is their high biodegradability and biocompatibility, as films were found to be non-toxic in cell viability assay. Overall, it can be concluded that AX-based films are potential candidates for application as wound dressings. However, to further improve patient compliance, the antibiotic release time of the films should be increased by the cross-linking of AX or incorporation of the other biopolymers in the film formulations.

## Figures and Tables

**Table 1:** Composition of the gels used to cast blank and gentamicin (GM)-loaded films.

Film Code	Arabinoxylan (% w/v)	Glycerol (% w/v)	Gentamicin (% w/v)	Water (q·s·mL)	Gel Weight (g/film)
AXF	2	–	–	100	33
AXF21	2	1	–	100	33
AXF1	1	2	–	100	33
AXF1.5	1.5	2	–	100	33
AXF2	2	2	–	100	33
AXF2.5	2.5	2	–	100	33
AXF3	3	2	–	100	33
AXF3.5	3.5	2	–	100	33
AXDF2	2	2	0.1	100	33
AXDF2.5	2.5	2	0.1	100	33
AXDF3	3	2	0.1	100	33

**Table 2:** Physical assessment of arabinoxylan (AX)-based films.

Film Code	Peelablity	Transparency	Bubbles	Flexibility	Interpretation
AXF	×	√	√	×	**Rejected:** thin, brittle, non-peelable, non-flexible films
AXF21	×	√	×	×	**Rejected:** rigid films, difficult to peel
AXF1	×	√	×	√	**Rejected:** thin, non-peelable, torn during peeling
AXF1.5	×	√	×	√	**Rejected:** thin, non-peelable, torn during peeling
AXF2	√	√	×	√	**Selected:** transparent, easily peelable, and flexible films
AXF2.5	√	√	×	√	**Selected:** transparent, easily peelable, and flexible films
AXF3	√	√	×	√	**Selected:** transparent, easily peelable, and flexible films
AXF3.5	√	×	√	√	**Rejected:** form viscous gel, difficult to pour and stir, uneven thickness, less transparent, bubbles

**Table 3:** Thickness, solvent loss, and mechanical characterization of films (mean ± SD, n =3). WVTR: water vapor transmission.

Films Code	Thickness (mm)	Solvent Loss (%)	Tensile Strength (N/mm2)	Elongation at Break (%)	WVTR (g/m^2^/day)
**AXF2**	0.309 ± 0.007	96.5 ± 1.4	Film damaged		1915 ± 135
**AXF2.5**	0.312 ± 0.008	96.1 ± 1.7	3.17 ± 0.14 ^#^	95.1 ± 6.3 ^#^	1747 ± 121
**AXF3**	0.331 ± 0.005 ^#^	95.2 ± 2.1	3.75 ± 0.21 ^#^	83.7 ± 4.6 ^#^	1757 ± 130
**AXDF2**	0.333 ± 0.007 *^×^	94.9 ± 2.3	Film damaged		1842 ± 170
**AXDF2.5**	0.354 ± 0.004 *^×^	92.3 ± 1.3	3.31 ± 0.17 ^×^	97.3 ± 3.4 ^×^	1631 ± 130
**AXDF3**	0.356 ± 0.006 *^×^	91.7 ± 2.2	3.79 ± 0.19 ^×^	84.9 ± 5.7 ^×^	1665 ± 155

The asterisks (*) show the significant difference of GM-loaded films from corresponding blank films, the hashes (^#^) show significant difference among blank films, and the crosses (^×^) show significant difference among GM-loaded films.

**Table 4:** Mathematical modeling of drug release from GM-loaded AX films.

Films	Zero Order		First Order		Higuchi		Korsmeyer–Peppas			Hixson–Crowell	
	R^2^	K_0_	R^2^	K_1_	R^2^	K	R^2^	K	n	R^2^	K
AXFD2.5	0.614	2.246	0.799	−0.037	0.819	14.63	0.911	1.565	0.511	0.615	−0.749
AXFD3	0.685	2.901	0.896	−0.044	0.875	18.50	0.979	1.405	0.651	0.685	−0.967

**Table 5:** Diameters of the zone of inhibitions (ZOIs) (mean ± SD, n = 3).

Sample	*S. aureus* (ZOI mm)	*P. aeruginosa* (ZOI mm)	*E. coli* (ZOI mm)
Blank Disk	9.3 ± 1.6	6.6 ± 0.8	6.4 ± 2.3
Blank Film	20.6 ± 1.2 *	11.2 ± 0.8 *	10.9 ± 1.2
GM Standard	31.8 ± 1.5 *^#^	27.5 ± 1.6 *^#^	21.9 ± 2.1 *^#^
AXFD2	34.7 ± 1.7 *^#^	20.1 ± 1.1 *^#×^	21.7 ± 2.2 *^#^
AXFD2.5	31.2 ± 1.9 *^#^	19.9 ± 1.2 *^#×^	21.1 ± 1.5 *^#^
AXFD3	30.3 ± 1.3 *^#^	23.3 ± 1.4 *^#^	21.1 ± 1.7 *^#^

The asterisk (*), hash (^#^) and cross (^×^) signs indicate a statistically significant difference of GM-loaded films from blank disks, blank films, and the GM standard, respectively.

## Supplementary Materials

The following are available online at https://www.mdpi.com/2073-4360/12/3/548/s1, Figure S1: Thickness of AX Films (mean ± SD, n = 3), Figure S2: Solvent (water) loss (%) during AX film casting (mean ± SD, n = 3), Table S1: TGA steps of the pure AX, blank AX films, and GM-loaded pAX films.

## References

[1] Pawar H.V., Boateng J.S., Ayensu I., Tetteh J. (2014). Multifunctional medicated lyophilised wafer dressing for effective chronic wound healing. J. Pharm. Sci..

[2] Simões D., Miguel S.P., Ribeiro M.P., Coutinho P., Mendonça A.G., Correia I.J. (2018). Recent advances on antimicrobial wound dressing: A review. Eur. J. Pharm. Biopharm..

[3] Boateng J.S., Matthews K.H., Stevens H.N.E., Eccleston G.M. (2008). Wound Healing Dressings and Drug Delivery Systems: A Review. J. Pharm. Sci..

[4] Singh B., Sharma S., Dhiman A. (2013). Design of antibiotic containing hydrogel wound dressings: Biomedical properties and histological study of wound healing. Int. J. Pharm..

[5] Patel S., Srivastava S., Singh M.R., Singh D. (2018). Preparation and optimization of chitosan-gelatin films for sustained delivery of lupeol for wound healing. Int. J. Biol. Macromol..

[6] Huang Y.-C., Huang K.-Y., Lew W.-Z., Fan K.-H., Chang W.-J., Huang H.-M. (2019). Gamma-irradiation-prepared low molecular weight hyaluronic acid promotes skin wound healing. Polymers.

[7] Kirketerp-Møller K., Zulkowski K., James G. (2011). Chronic wound colonization, infection, and biofilms. Biofilm infections.

[8] Phan T.T.V., Huynh T.-C., Oh J. (2019). Photothermal Responsive Porous Membrane for Treatment of Infected Wound. Polymers.

[9] Ng S.-F., Leow H.-L. (2015). Development of biofilm-targeted antimicrobial wound dressing for the treatment of chronic wound infections. Drug Dev. Ind. Pharm..

[10] Said J., Dodoo C.C., Walker M., Parsons D., Stapleton P., Beezer A.E., Gaisford S. (2014). An in vitro test of the efficacy of silver-containing wound dressings against Staphylococcus aureus and Pseudomonas aeruginosa in simulated wound fluid. Int. J. Pharm..

[11] Bendy J.R., Nuccio P., Wolfe E., Collins B., Tamburro C., Glass W., Martin C. (1964). Relationship of quantitative wound bacterial counts to healing of decubiti: Effect of topical gentamicin. Antimicrob. Agents Chemother..

[12] Boateng J.S., Pawar H.V., Tetteh J. (2013). Polyox and carrageenan based composite film dressing containing anti-microbial and anti-inflammatory drugs for effective wound healing. Int. J. Pharm..

[13] Alavi T., Rezvanian M., Ahmad N., Mohamad N., Ng S.-F. (2019). Pluronic-F127 composite film loaded with erythromycin for wound application: Formulation, physicomechanical and in vitro evaluations. Drug Deliv. Transl. Res..

[14] Bowler P., Duerden B., Armstrong D.G. (2001). Wound microbiology and associated approaches to wound management. Clin. Microbiol. Rev..

[15] Pawar H., Tetteh J., Boateng J. (2013). Preparation, optimisation and characterisation of novel wound healing film dressings loaded with streptomycin and diclofenac. Colloids Surf. B.

[16] Naseri-Nosar M., Ziora Z.M. (2018). Wound dressings from naturally-occurring polymers: A review on homopolysaccharide-based composites. Carbohydr. Polym..

[17] Farzamfar S., Naseri-Nosar M., Samadian H., Mahakizadeh S., Tajerian R., Rahmati M., Vaez A., Salehi M. (2018). Taurine-loaded poly (ε-caprolactone)/gelatin electrospun mat as a potential wound dressing material: In vitro and in vivo evaluation. J. Bioact. Compat. Polym..

[18] Dhivya S., Padma V.V., Santhini E. (2015). Wound dressings—A review. BioMedicine.

[19] Kamoun E.A., Kenawy E.-R.S., Chen X. (2017). A review on polymeric hydrogel membranes for wound dressing applications: PVA-based hydrogel dressings. J. Adv. Res..

[20] Rezvanian M., Amin M.C.I.M., Ng S.-F. (2016). Development and physicochemical characterization of alginate composite film loaded with simvastatin as a potential wound dressing. Carbohydr. Polym..

[21] Olewnik-Kruszkowska E., Gierszewska M., Jakubowska E., Tarach I., Sedlarik V., Pummerova M. (2019). Antibacterial Films Based on PVA and PVA–Chitosan Modified with Poly (Hexamethylene Guanidine). Polymers.

[22] Rezk A.I., Lee J.Y., Son B.C., Park C.H., Kim C.S. (2019). Bi-layered Nanofibers Membrane Loaded with Titanium Oxide and Tetracycline as Controlled Drug Delivery System for Wound Dressing Applications. Polymers.

[23] Peng X.-w., Ren J.-l., Zhong L.-x., Sun R.-c. (2011). Nanocomposite films based on xylan-rich hemicelluloses and cellulose nanofibers with enhanced mechanical properties. Biomacromolecules.

[24] Massey S., Iqbal M.S., Wolf B., Mariam I., Rao S. (2016). Comparative drug loading and release study on some carbohydrate polymers. Lat. Am. J. Pharm..

[25] Amin M., Iram F., Iqbal M.S., Saeed M.Z., Raza M., Alam S. (2013). Arabinoxylan-mediated synthesis of gold and silver nanoparticles having exceptional high stability. Carbohydr. Polym..

[26] Akbar J., Iqbal M.S., Massey S., Masih R. (2012). Kinetics and mechanism of thermal degradation of pentose-and hexose-based carbohydrate polymers. Carbohydr. Polym..

[27] Akbar J., Iqbal M.S., Chaudhary M.T., Yasin T., Massey S. (2012). A QSPR study of drug release from an arabinoxylan using ab initio optimization and neural networks. Carbohydr. Polym..

[28] Bhatia M., Ahuja M. (2015). Psyllium arabinoxylan: Carboxymethylation, characterization and evaluation for nanoparticulate drug delivery. Int. J. Biol. Macromol..

[29] Erum A., Bashir S., Saghir S., Tulain U.R., Saleem U., Nasir M., Kanwal F., Hayat malik M.N. (2015). Acute toxicity studies of a novel excipient arabinoxylan isolated from Ispaghula (Plantago ovata) husk. Drug Chem. Toxicol..

[30] Saghir S., Iqbal M.S., Koschella A., Heinze T. (2009). Ethylation of arabinoxylan from Ispaghula (Plantago ovata) seed husk. Carbohydr. Polym..

[31] Iqbal M.S., Akbar J., Hussain M.A., Saghir S., Sher M. (2011). Evaluation of hot-water extracted arabinoxylans from ispaghula seeds as drug carriers. Carbohydr. Polym..

[32] Saghir S., Iqbal M.S., Hussain M.A., Koschella A., Heinze T. (2008). Structure characterization and carboxymethylation of arabinoxylan isolated from Ispaghula (Plantago ovata) seed husk. Carbohydr. Polym..

[33] Rezvanain M., Ahmad N., Amin M.C.I.M., Ng S.-F. (2017). Optimization, characterization, and in vitro assessment of alginate-pectin ionic cross-linked hydrogel film for wound dressing applications. Int. J. Biol. Macromol..

[34] Matthews K., Stevens H., Auffret A., Humphrey M., Eccleston G. (2005). Lyophilised wafers as a drug delivery system for wound healing containing methylcellulose as a viscosity modifier. Int. J. Pharm..

[35] Bauer A., Kirby W., Sherris J.C., Turck M. (1966). Antibiotic susceptibility testing by a standardized single disk method. Am. J. Clin. Pathol..

[36] Sionkowska A., Kaczmarek B., Gadzala-Kopciuch R. (2016). Gentamicin release from chitosan and collagen composites. J. Drug Deliv. Sci. Technol..

[37] Kachel-Jakubowska M., Matwijczuk A., Gagoś M. (2017). Analysis of the physicochemical properties of post-manufacturing waste derived from production of methyl esters from rapeseed oil. Int. Agrophys..

[38] Perotti G.F., Kijchavengkul T., Auras R.A., Constantino V.R. (2017). Nanocomposites Based on Cassava Starch and Chitosan-Modified Clay: Physico-Mechanical Properties and Biodegradability in Simulated Compost Soil. J. Braz. Chem. Soc..

[39] Iqbal M.S., Akbar J., Saghir S., Karim A., Koschella A., Heinze T., Sher M. (2011). Thermal studies of plant carbohydrate polymer hydrogels. Carbohydr. Polym..

[40] Almazrouei M., Samad T.E., Janajreh I. (2017). Thermogravimetric Kinetics and High Fidelity Analysis of Crude Glycerol. Energy Procedia.

[41] Korsmeyer R.W., Gurny R., Doelker E., Buri P., Peppas N.A. (1983). Mechanisms of solute release from porous hydrophilic polymers. Int. J. Pharm..

[42] Ritger P.L., Peppas N.A. (1987). A simple equation for description of solute release II. Fickian and anomalous release from swellable devices. J. Control. Release.

[43] Erum A., Bashir S., Saghir S. (2015). Modified and unmodified arabinoxylans from Plantago ovata husk: Novel excipients with antimicrobial potential. Bangladesh J. Pharmacol..

[44] (2009). ISO I. 10993–5: 2009 Biological Evaluation of Medical Devices–Part 5: Tests for In Vitro Cytotoxicity.

