# Supplementary material for: Development and Characterization of Hemicellulose-Based Films for Antibacterial Wound-Dressing Application
Source: Polymers (Basel). 2020 Mar 3;12(3):548. doi: 10.3390/polym12030548 (PMC7183054; doi:10.3390/polym12030548)
Supplement: Supplementary file 1 [file polymers-12-00548-s001.pdf]

polymers

Supplementary Information: Development and Characterization of Hemicellulose Based Films for Antibacterial Wound Dressing Application

Naveed Ahmad [1,2,*], Danial Tayyeb [3], Imran Ali [3], Nabil K. Alruwaili [1], Waqas Ahmad [4], Atta ur Rehman [2], Abdul Haleem Khan [2] and Mohammad Saeed Iqbal [3,*]

[1] Department of Pharmaceutics, College of Pharmacy, Jouf University, Sakaka, Aljouf 72388, Saudi Arabia
[2] Department of Pharmacy, Forman Christian College, Ferozepur road, Lahore 54600, Pakistan
[3] Department of Chemistry, Forman Christian College, Ferozepur road, Lahore 54600, Pakistan
[4] School of Pharmaceutical Sciences, University of Science Malaysia, Pulau Pinang 11800, Malaysia
* Correspondence: saeediqbal@fccollege.edu.pk (M.S.I.); naveedpharmacist@yahoo.com (N.A.)

Received: 27 December 2019; Accepted: 17 February 2020; Published: date

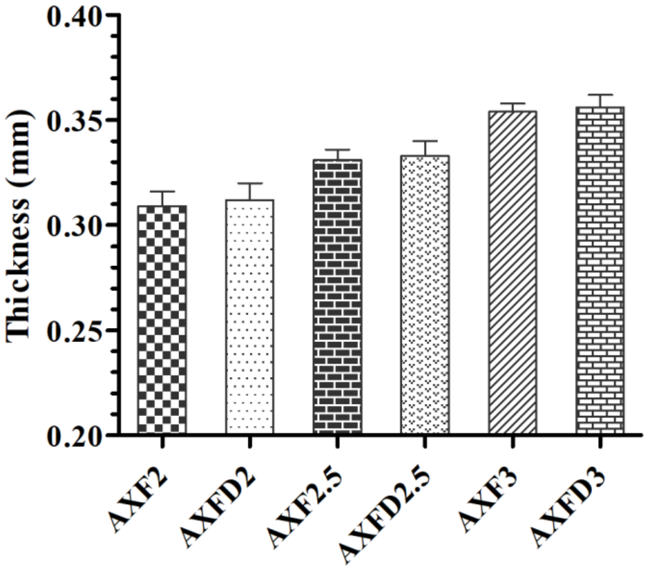

Figure S1. Thickness of AX Films (mean ± SD, n = 3).

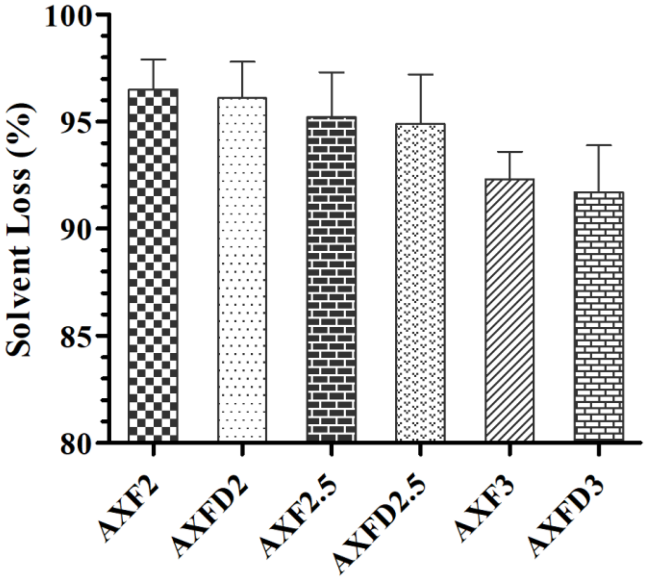

Figure S2. Solvent (water) loss (%) during AX film casting (mean ± SD, n = 3).

Table S1. TGA steps of the pure AX, blank AX films and GM-loaded pAX films.

Sample	Step I		Step II		Step III		Residue	
	**Temp. (°C)**	**Wt. Loss (%)**	**Temp. (°C)**	**Wt. Loss (%)**	**Temp. (°C)**	**Wt. Loss (%)**	**Temp. (°C)**	**Wt. (%)**
**AX**	<120	11.5	230 to 335	40.5	>410	12.2	500	28.3
**AXF2**	<110	16.6	160 to 260	42.3	>310	16.2	450	12.4
**AXDF2**	<110	15.6	160 to 260	51.1	>310	16.5	450	3.3
**AXF2.5**	<120	14	180 to 260	50.3	>310	11.9	450	10.3
**AXDF2.5**	<120	11.5	180 to 260	43.7	>310	11.6	450	13.6
**AXF3**	<120	13.5	185 to 270	38.4	>315	10.5	450	20.5
**AXDF3**	<120	13.9	185 to 270	41.3	>315	11.3	450	16.6
